# Study on the influence of urban tree canopy on thermal environment in Luoping County

**DOI:** 10.1038/s41598-023-40449-2

**Published:** 2023-08-16

**Authors:** Ji-ping Dai, Ji-zheng Qin, Tianyu Zhou, Xiao-jian Qin, Kun Zhu, Jian-song Peng

**Affiliations:** 1 Southwest Forestry University, Kunming, 650224 Yunnan China; 2 National Forestry and Grassland Bureau Survey Planning and Design Institute, Beijing, 100714 China; 3 Kunming Science and Technology Fanya Design Group Co., LTD, Kunming, 650224 Yunnan China

**Keywords:** Ecology, Environmental sciences

## Abstract

Urban forest is an integral part of the complex urban ecosystem, and tree canopy plays a key role in improving urban climatic environment. Urban Tree Canopy (UTC) is strongly linked to urban thermal environment and living quality of residents. In this study, Luoping County, a mountainous county in southwest China, was selected as the study area to uncover the inner connections between tree canopy and thermal environment, and provide relevant scientific references for the construction of livable forest cities in similar areas. Through eCongnition Developer, ENVI and ArcGIS software, the distribution of Land Surface Temperature (LST) and land cover types in the study area was extracted, 63 patches with super-large and extra-large tree canopy coverage selected, to explore the regulatory effect of UTC patches on urban thermal environment based on SPSS software. Results showed that the highest LST in the research area was 37.63 ℃, the lowest 24.73 ℃, and the average 30.83 ℃. Among the land cover types, the area of buildings and impervious surfaces was 1615.71 hm^2^, accounting for 55.76% of the total study area, which was the largest proportion and with widespread distribution; the area of grassland and water body was 57.48 hm^2^ and 12.35 hm^2^, respectively, taking up 1.98% and 0.43%, with a smaller proportion. Mean LST: impervious surface > bare land > grassland > tree canopy > water body. By increasing the area and perimeter of the patch covered by tree canopy, the cooling rate of the patch can be increased while the temperature inside the patch can be reduced. The relationship between the area and cooling rate is closer than that between perimeter and cooling rate. The increase of perimeter has a stronger alleviation effect on the internal temperature of the patch, whereas, the increase of area has a weaker effect in this respect.

## Introduction

Along with urban expansion and development, a series of issues emerged, such as the increase in impervious surface in roads and buildings, the decrease in green space and water body, the intensification of landscape fragmentation, and the rise of anthropogenic heat emission, which led to the gradual deterioration of urban thermal environment, imposing an adverse effect on people’s production and life^1–3^. And Urban Heat Island (UHI) has become a common environmental problem faced by cities today, which drew the attention of multidisciplinary scholars at home and abroad as to how to alleviate the impact of UHI^4–7^. Correspondingly, urban forest, as a significant part of the complex urban ecosystem with self-purification function, has developed rapidly. UTC, a special urban landscape, is the expression of urban forest at a smaller scale in the landscape, which plays an important role in improving urban climatic environment, supporting smart planning and evaluating the quality of ecosystem services^8–11^. Hence, there is a high correlation between UTC and urban thermal environment and residents’ living quality^12^.

At present, the study of thermal environment at home and abroad majorly focused on the thermal environment effect based on urban land use, urban landscape, and the spatial distribution of urban green space, type and pattern. There have been some researches targeted UTC, nevertheless, the research work is by no means profound, limited to the metropolises such as Beijing, Shanghai, Guangzhou and Shenzhen. And studies on the forest vertical differentiation in southwest China, plateau and mountain cities with diverse forest vegetation types are just a few. This study aims at deeply exploring the internal relationship between UTC and thermal environment, by selecting the mountainous city in southwest China as the study area, and utilizing the multidisciplinary knowledge of geographic information system, landscape ecology, environmental science, statistics and so on in a comprehensive manner, in the hope of providing relevant scientific references for the establishment of livable forest cities in similar regions.

## Overview of the study area

**Figure 1:**
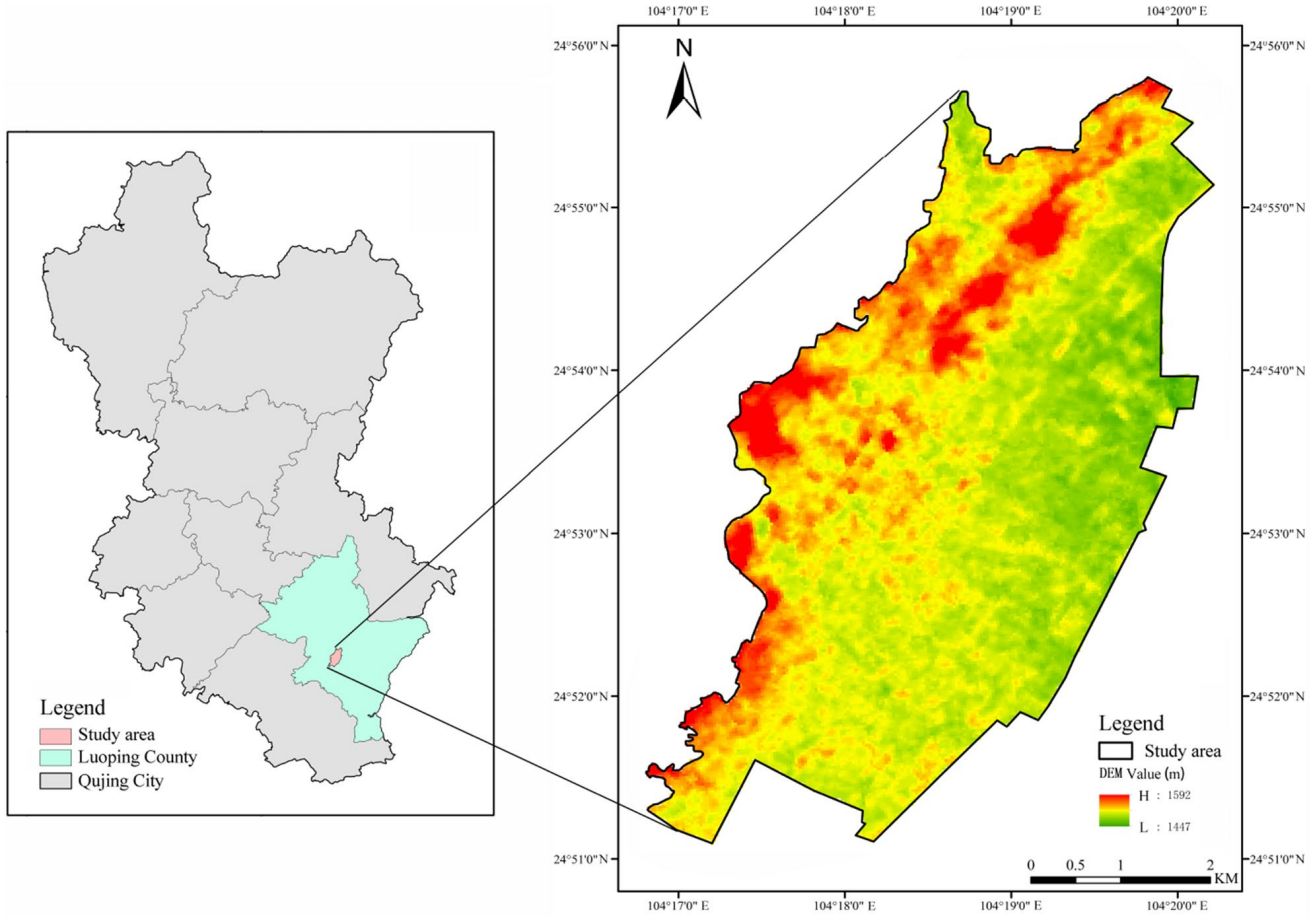
Location of the Study Area, the software used to create the map is Arcgis 10.7 (https://www.esri.com/zh-cn/arcgis/products/arcgis-desktop/resources).

Qujing City, awarded the “National Forest City” in November 2019, is a typical highland mountain forest city. Luoping County, Qujing City, at east longitude 103°57′–104°43′ and north latitude 24°31′–25°25′, is located at the juncture of Yunnan, Guangxi and Guizhou provinces (regions), with convenient transportation, beautiful scenery, livable conditions, and abundant natural resources. The forest coverage rate stands at 46.51%, average annual temperature 15.1 ℃ and good air quality rate 100% all year long, and its negative oxygen ion concentration is as high as 20,000 per cubic centimeter. Luoping County boasts outstanding advantages in terms of location, climate, ecology and resources. It is also a part of the optimized development zone of forest city in central Yunnan Province, which makes the selection of the County as the research site both typical and representative. Moreover, with obvious UHI effect, its urban built-up area is a “natural laboratory” for studying environmental changes^13^. Therefore, this study takes the urban built-up area of Luoping County as the study area, administrative division from the BIGEMAP website (http://www.bigemap.com/), DEM data from the Geospatial Data Cloud platform (https://www.gscloud.cn/ (Grids at 30 m resolution)) (Fig. 1). Up to 2020, the study area is 2897.7 hm^2^, tree canopy planimetric area 731.37 hm^2^, and tree canopy coverage 25.24%.

## Data and methods

### Technical process

**Figure 2:**
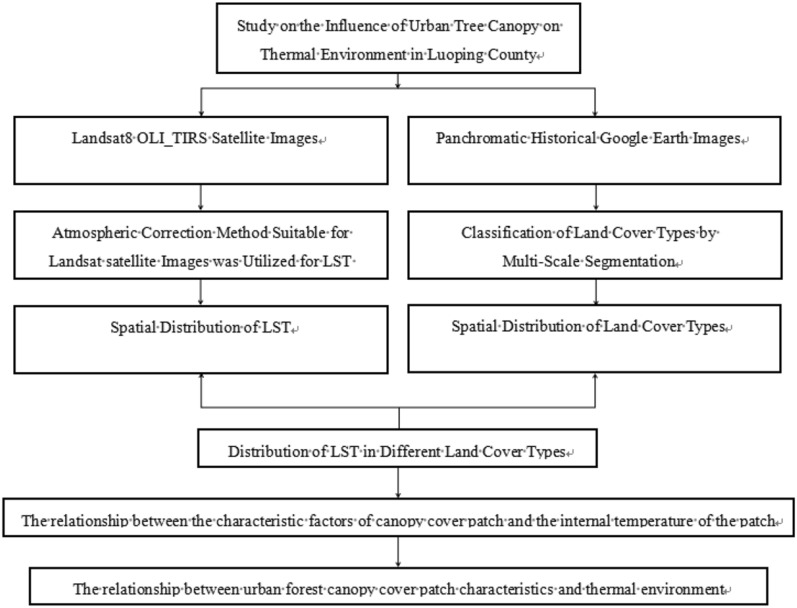
Technical flowchart.

In this study, through eCongnition Developer, ENVI and ArcGIS software, the distribution of Land Surface Temperature (LST) and land cover types in the study area was extracted, 63 patches with super-large and extra-large tree canopy coverage selected, to explore the regulatory effect of UTC patches on urban thermal environment based on SPSS software (Fig. 2).

### LST inversion

LST inversion was carried out on Landsat8OLI_TIRS satellite images (https://www.gscloud.cn/) by means of ENVI5.3 software. Commonly used LST inversion algorithms mainly include atmospheric correction method, split-window algorithm and single-channel algorithm. In this study, atmospheric correction method suitable for Landsat satellite images was utilized for LST inversion (Table 1)^14–16^. The principle is to first estimate the influence of atmosphere on surface thermal radiation, and then subtract this part of atmospheric influence from the total thermal radiation observed by satellite sensors to obtain surface thermal radiation intensity, and then convert it into LST^17^.

**Table 1:** Expression of LST inversion based on atmospheric correction method.

Expression	Parameter
Thermal infrared radiance $L_{\lambda } = \left[ {\varepsilon B\left( {T_{S} } \right) + \left( {1 - \varepsilon } \right)L \downarrow } \right]\tau + L \uparrow$	Input imaging time (2021-03-18, 3:28), central longitude (Lon: 104.6715), central latitude (Lat: 24.5471) in the US website (http://atmcorr.gsfc.nasa.gov) released by NASA, atmospheric profile: τ = 0.91, L↑ = 0.75 W/m^2^/sr/μm, L↓ = 1.29 W/m^2^/sr/μm
Black body thermal radiance $B\left( {T_{S} } \right) = \frac{{L_{\lambda } - L \uparrow - \tau \left( {1 - \varepsilon } \right)L \downarrow }}{\tau \varepsilon }$	
Real LST $T_{S} = \frac{{K_{2} }}{{\ln \left( {K_{1} /B\left( {T_{S} } \right) + 1} \right)}}$	K1 and K2: calibration constants, for TIRS Band10, $K_{1} = 774.89\;{\text{W}}/\left( {{\text{m}}^{2 * } \;{\mu m}^{ * } {\text{sr}}} \right)$, $K_{2} = 1321.08\;{\text{K}}$
Surface specific emissivity $\varepsilon = 0.004P_{V} + 0.986$	–
Vegetation coverage $P_{V} = \frac{NDVI - NDVISoil}{{NDVIeg - NDVIS{\text{o}}il}}$	NDVI: normalized vegetation index; NDVISoil: NDVI value of the bare land or the area without vegetation; NDVIVeg: NDVI value of the area completely covered by vegetation

The image was obtained on March 18th, 2021, row number 128/43, and resolution 30 m. The cloudage was low (0.01) at the time of image acquisition, thus the image quality was high, with the ground objects being clearly displayed. In consequence, the retrieved temperature correctly manifested the urban LST.

### Extraction of land cover types

With the help of eCognition Developer 9.0 object-oriented classification software, the panchromatic historical Google Earth images (https://www.google.com/earth/ (Grids at 0.54 m resolution in 2020)) in January 2020 was interpreted and processed. ECognition software owns a variety of built-in segmentation algorithms, each of which has its own merits and demerits^18,19^. In this study, a multi-scale segmentation method, which calls for high boundary accuracy, and the image objects being close to the natural boundary of ground objects, was adopted for image segmentation. As it was of great significance to set appropriate segmentation parameters, the optimal segmentation parameters of heterogeneous patches were obtained (Table 2) through constant adjustment and improvement. The land cover types in the study area were split into five categories: water body, tree canopy, bare land, grassland, and impervious surface, based on which overlay analysis was performed in combination with LST, and the contribution degree of each land cover type to urban thermal environment was studied.

**Table 2:** Optimal segmentation parameter of landscape patch.

Level	Scale parameter	Color parameter	Compactness parameter	Object of classification	
1	180	0.4	0.7	Water body	
2	20	0.6	0.8	Tree canopy	Single tree and shrub
3	60	0.4	0.7		Multiple trees and shrubs
4	120	0.5	0.7	Bare land and impervious surface	
5	30	0.4	0.7	Grassland	

The tree canopy patches were classified on the basis of the classification method of tree canopy patch by Wu et al.^20^ and Yao et al.^21^, and the current situation of tree canopy patch area in the study area. Small patch ≤ 0.05 hm^2^, 0.05 hm^2^ < Medium-sized patch ≤ 0.20 hm^2^, 0.20 hm^2^ < large patch ≤ 1.00 hm^2^, 1.00 hm^2^ < super-large patch ≤ 5.00 hm^2^, extra-large patch > 5.00 hm^2^.

### Calculation of Tree Canopy patch shape index and cooling rate

In order to eliminate the influence of environmental gradient change on LST, the cooling rate was chosen as the performance factor to mitigate the heat island effect. According to the research of Cui^23^ and Li et al.^22^, the shape index and cooling rate of tree canopy patches were calculated. The formula is as follows:

1 $$ {\text{Patch}}\;{\text{ shape}}\;{\text{ index}}\;\;A = \frac{E}{{2\sqrt {\pi S} }} $$

where, E represents patch perimeter; S represents patch area.

2 $$ {\text{Cooling}}\;{\text{ rate}}/\% \; = \;({\text{Mean}}\;{\text{ LST}}\;{\text{of }}\;{\text{bare}}\;{\text{ land}}\; \, - \;{\text{Mean }}\;{\text{LST }}\;{\text{of}}\;{\text{ tree}}\;{\text{ canopy}}\;{\text{ patch}})/{\text{Mean }}\;{\text{LST}}\;{\text{ of }}\;{\text{bare }}\;{\text{land}} $$

## Results and analysis

### Spatial distribution features of LST

**Figure 3:**
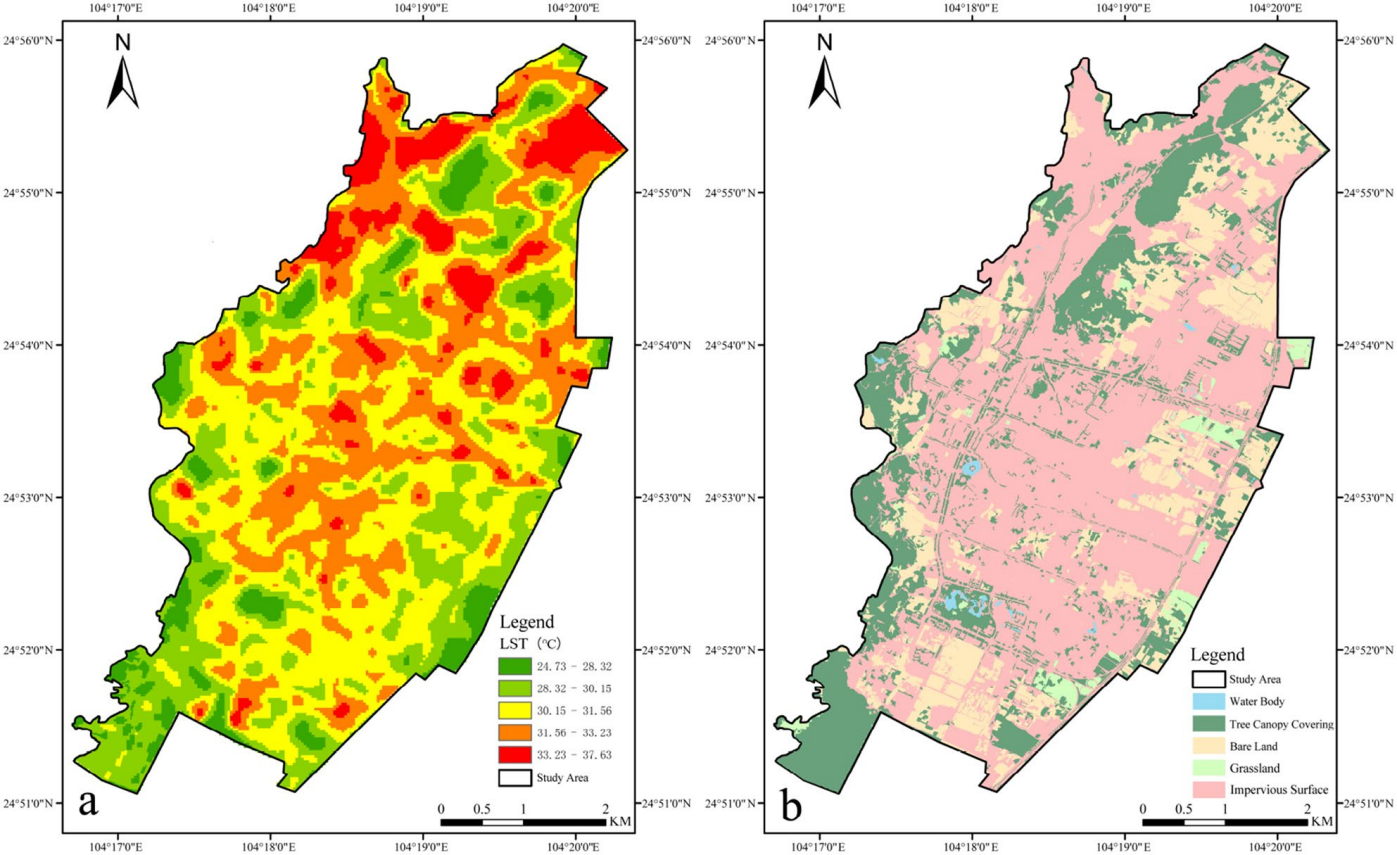
Map of LST Classification, the software used to create the map is ENVI 5.3 (https://envi.geoscene.cn/) & Land Cover Type in the Study Area, the software used to create the map is eCognition Developer 9.0 (https://geospatial.trimble.com/ecognition-trial).

Based on LST inversion (Fig. 3a), LST in the study area ranged from 24.73 to 37.63 ℃, and the mean LST was 30.8 ℃. In order to maximize the difference in data values between temperature levels, according to the natural breaks classification method of Arcgis 10.7 in the study, the temperature data was divided into five levels: low temperature, mild low temperature, medium temperature, sub-high temperature and high temperature, temperature range of 24.73–28.32 ℃, 28.32–30.15 ℃, 30.15–31.56 ℃, 31.56–33.23 ℃, and 33.23–37.63 ℃. The high temperature area was mainly distributed in the northern part of the study area with less vegetation coverage, which was a large area of impervious surface and bare land, and the other parts showed sporadic distribution. The sub-high temperature area was found in the vicinity of the high temperature area, majorly impervious surface and bare land. Low temperature area was primarily in large areas of park green spaces, water bodies and natural mountains and areas with high tree canopy coverage, such as Liangmashan Forest Park, Xiangshijie Forest Park, Mass Culture Park and other areas, which were conducive to reducing the UHI effect. The sub-low temperature area was around the low temperature area, mainly the area with high tree canopy coverage. The rest were medium temperature regions, whose wide distribution in the whole study area was relatively uniform. On the whole, LST in the northern region was higher than that in the southern region.

### Distribution features of land cover types

In the light of the extraction of land cover types (Fig. 3b), the area of buildings and impervious surface in the study area was 1615.71 hm^2^, accounting for 55.76% of the total study area, which took up the largest proportion and was widely distributed when compared with other land cover types. The tree canopy covering area was 731.37 hm^2^, occupying 25.24% of the total, among which the larger tree canopy patches were mainly found in the northern, southern and western edges of the study area, such as Liangmashan Forest Park, Xiangshiji Forest Park, and Mass Culture Park and other park green spaces, as well as natural mountains. The smaller tree canopy patches in the central, southern and eastern parts of the study area were scattered, principally including roadside green spaces such as Wanfeng Road, Longmen Street, Songgang Road, Huansong Street, Huahai Avenue and Lubuge Avenue, petty street gardens such as Wenbi Road, Sanjiang Street and Gold Coast Huating, and road green spaces such as Xiguan Street, Longmen Street, Zhenxing Street, Wenbi Road, Kowloon Avenue, Huahai Avenue, Lubuge Avenue, Yongkang Road and Wanfeng Road etc. In the case of bare land, the acreage was 480.79 hm^2^, representing 16.59% of the total, mainly distributed in the northern, southern, eastern and western edges, which was the bare land and temporarily idle land during the construction of building lot. The grassland of 57.48 hm^2^ and water body of 12.35 hm^2^ occupied a smaller proportion, 1.98% and 0.43%, respectively. The sporadic distribution of grassland in the study area was majorly grassland in the urban green space and grassland temporarily covered by some vacant lands. The main water bodies were Mass Culture Park, Lushan Lake Park, Taiye Lake Park and other parks.

### Distribution features of LST in different land cover types

**Figure 4:**
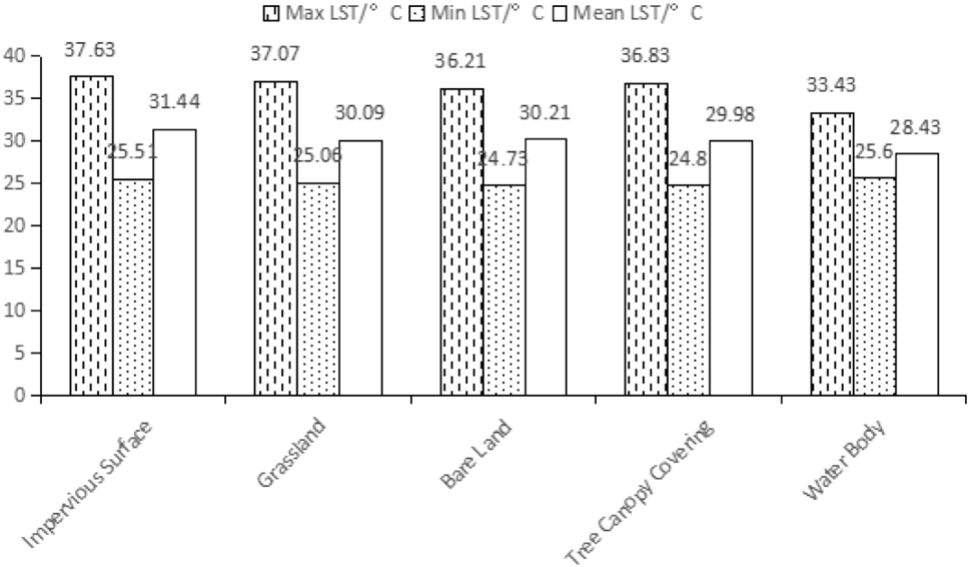
LST statistics of different land cover types.

Based on the superposition of different land cover types and LST by Arcgis 10.7 (Fig. 4), mean LST of different cover types was significantly different, impervious surface (31.44 ℃) > bare land (30.21 ℃) > grassland (30.09 ℃) > tree canopy (29.98 ℃) > water body (28.43 ℃). The mean LST of bare land, grassland, tree canopy and water body was lower than that of the study area, whereas the mean LST of the impervious surface was higher in this regard. The mean LST of impervious surface was the highest, contributing the most to the heat island effect, which was primarily related to surface materials such as concrete and asphalt, anthropogenic heat sources and a great deal of energy consumption. Grassland did not have a distinct cooling effect. The mean LST of water body was the lowest, principally on account of its large specific heat capacity and low thermal emissivity, which could effectively reduce sensible heat exchange capacity, thus playing a crucial role in ameliorating local microclimate and urban thermal environment. The transpiration and low surface emissivity of trees made its tree canopy have a cooling effect on LST. However, due to the small area, large edge ratio and scattered distribution of some patches, some patches were easily to be influenced by the surrounding environment, displaying strong heat exchange, and leading to the higher average temperature compared with the water body.

### Spatial pattern analysis of tree canopy

The extraction of tree canopy (Table 3) indicated that there were 1432 tree canopy patches in the study area, among which 492 patches were small, 579 patches medium-sized, 298 patches large, 48 patches super-large and 15 patches extra-large. The number of giant patches with area > 5 hm^2^ was small, while the patch area took up 59.91%, the largest proportion of the total patch area. The number of medium-sized patches with 0.05 hm^2^ < area ≤ 0.20 hm^2^ was the largest, while the proportion of patch area was relatively small, accounting for 8.74% of the total patch area. Small patch area ≤ 0.05 hm^2^ made up the smallest proportion, 1.95% of the total. The hierarchical statistics demonstrated that the super-large and extra-large patches of urban forest were mainly distributed in the northern, southern and western edges of the study area with low impact factors, while the large, medium-sized and small patches were relatively evenly distributed in the whole study area, principally in the central area. By comparing tree canopy coverage with the LST distribution in the study area, it could be seen that smaller patches manifested implicit heterogeneity and cooling effect. As a result, tree canopies with super-large and extra-large area, diverse shapes and salient cooling effect were selected to probe into the regulatory effect of UTC patches on urban thermal environment. Based on Arcgis 10.7, the area, perimeter and mean LST of 63 tree canopies with super-large and extra-large patches in the study area were extracted, and the patch shape index and cooling rate were calculated (Table 4). The date range of patch area and perimeter was broad. The maximum area of tree canopy patch area was 65.32 hm^2^, with a circumference of 25.17 km in Liangmashan Forest Park, followed by Qingshan Mountain, which surrounded the city, whose patch area was 64.54 hm^2^, 32.29 km in circumference. The minimum area was 1.01 hm^2^, 0.84 km in circumference, in the tree canopy beside Xiaoba Highway.

**Table 3:** Statistics of composition of tree canopy patch level.

Patch level	Patch quantity/unit	Patch area/hm^2^	Ratio of total patch area/%	Mean LST/°C
Small	492	14.27	1.95	31.39
Medium-sized	579	63.89	8.74	31.22
Large	298	122.68	16.77	30.72
Super-large	48	92.34	12.63	30.15
Extra-large	15	438.19	59.91	29.58
Total	1432	731.37	100	–

**Table 4:** Statistics of features of super-large and extra-large tree canopy patches.

Patch no	Patch area/hm^2^	Patch circumference/km	Patch shape index	Cooling rate/%
1	65.32	25.17	8.79	4.60
2	64.54	32.29	11.34	6.22
3	56.18	13.73	5.17	6.75
4	45.24	25.18	10.56	5.06
5	18.60	4.70	3.07	6.39
6	18.12	14.53	9.63	7.58
7	16.47	6.29	4.37	7.81
8	10.95	4.90	4.18	− 3.81
9	10.14	6.56	5.81	10.33
10	8.94	5.43	5.12	8.61
11	8.20	7.57	7.46	1.59
12	7.16	4.76	5.02	− 3.57
13	6.71	4.58	4.99	− 2.38
14	6.39	3.39	3.78	7.41
15	6.00	4.65	5.35	0.63
16	4.80	2.45	3.16	− 4.17
17	4.70	3.83	4.98	5.69
18	3.49	6.67	10.06	0.63
19	3.38	2.15	3.29	− 0.63
20	3.38	3.25	4.99	3.74
21	3.17	3.53	5.59	6.75
22	3.13	3.29	5.25	0.70
23	3.07	2.35	3.78	− 8.18
24	2.74	1.92	3.26	− 0.53
25	2.60	2.10	3.67	1.42
26	2.43	2.32	4.21	4.87
27	2.39	3.63	6.62	1.75
28	2.39	2.68	4.90	− 2.58
29	2.23	3.88	7.33	− 7.38
30	2.22	1.37	2.60	1.52
31	2.10	2.20	4.28	0.30
32	2.00	1.90	3.80	2.75
33	1.99	2.08	4.16	0.43
34	1.94	1.31	2.65	6.02
35	1.92	1.51	3.08	8.14
36	1.70	1.31	2.82	− 2.38
37	1.68	2.75	5.99	− 3.11
38	1.61	1.05	2.32	− 0.03
39	1.56	1.12	2.53	− 1.85
40	1.55	1.78	4.03	− 0.23
41	1.53	1.53	3.49	− 6.69
42	1.53	1.32	3.00	− 0.86
43	1.48	2.50	5.78	2.68
44	1.46	1.41	3.29	− 10.86
45	1.41	1.24	2.94	− 0.30
46	1.34	1.10	2.69	3.44
47	1.31	1.65	4.05	6.98
48	1.27	0.78	1.94	− 6.09
49	1.21	2.18	5.59	1.62
50	1.21	2.28	5.86	0.50
51	1.20	1.40	3.59	− 5.10
52	1.19	1.98	5.13	− 3.05
53	1.17	1.16	3.03	5.16
54	1.17	2.60	6.78	− 7.41
55	1.16	2.49	6.54	− 0.70
56	1.13	2.64	7.01	5.43
57	1.13	1.02	2.71	5.10
58	1.09	0.84	2.26	− 0.89
59	1.06	1.63	4.48	0.10
60	1.05	1.38	3.80	6.52
61	1.05	1.56	4.29	− 2.28
62	1.01	0.98	2.74	6.55
63	1.01	0.84	2.37	− 15.33

### Influence of tree canopy features on urban thermal environment

Based on SPSS 22.0 software, Pearson correlation analysis was conducted on the dependent variable—tree canopy patch cooling rate and the independent variable—tree canopy patch area, perimeter and shape index, respectively. The results of bivariate test displayed that the correlation coefficients between tree canopy patch area, perimeter and shape index, and patch cooling rate were *r* = 0.319, *r* = 0.313, *r* = 0.218, respectively, and the significance was *P* = 0.011 < 0.05, *P* = 0.013 < 0.05, *P* = 0.086 > 0.05, respectively. It served to show that patch area, perimeter and shape index were positively correlated with the cooling rate of patch, while the degree of significance was different for each group. Area, perimeter and cooling rate were significantly correlated at level 0.05, nevertheless, shape index and cooling rate were not significantly correlated at the same level. This indicated that by increasing the area and perimeter of tree canopy patch, the cooling rate of the patch could be improved, the internal temperature of the patch reduced.

**Figure 5:**
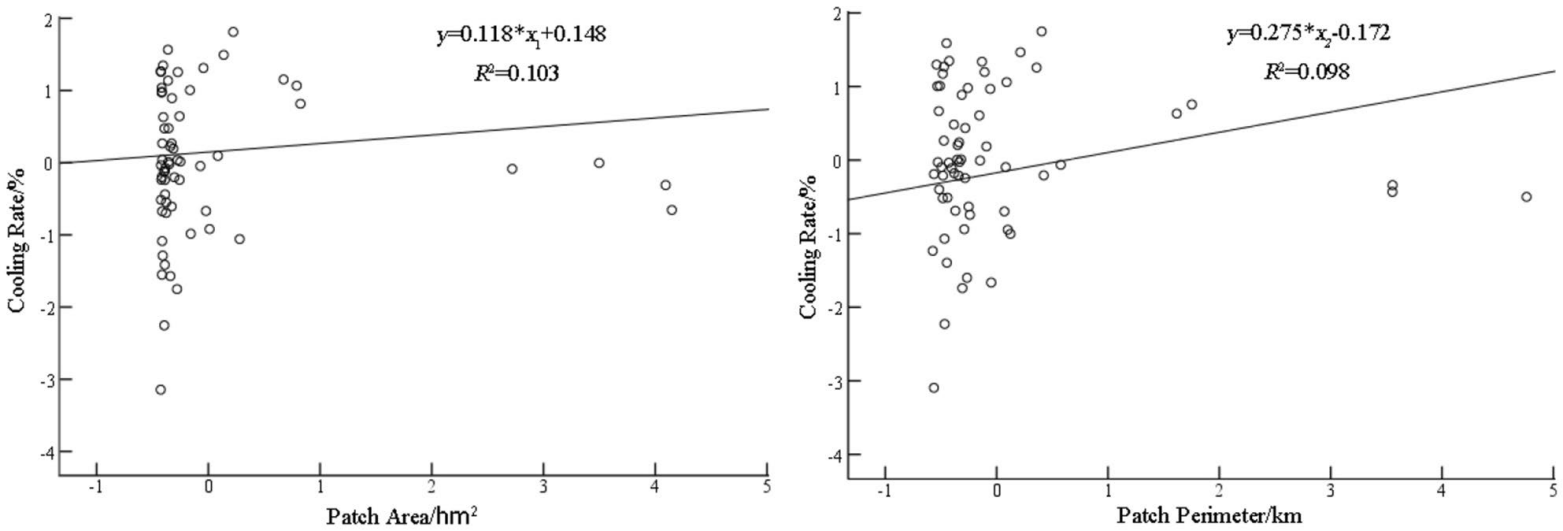
Linear regression analysis of tree canopy patch area, perimeter and cooling rate.

By the method of stepwise regression, the tree canopy patch shape index, which had the least impact on the significance of the model and was the least important independent variable, was removed from all the available independent variables to reduce the confounding interference among the models. Regression analysis was conducted on patch area (*x*_1_), perimeter (*x*_2_) and cooling rate (*y*), and the regression equation with optimal fitting accuracy was established (Fig. 5). The linear model regression equations were *y* = 0.118*x_1_ + 0.148, *y* = 0.275*x_2_−0.172, respectively. Durbin-watson values were 1.955 and 2.007, respectively, demonstrating no auto-correlation between patches. With regard to the determination coefficient (*R*^2^) of the regression equation, area and cooling rate, and perimeter and cooling rate were 0.103 and 0.098, respectively. The determination coefficient between area and cooling rate was relatively large, indicating that the relationship between area and cooling rate was closer than that between perimeter and cooling rate. The slope factor of the regression equation evinced that area and cooling rate, perimeter and cooling rate were 0.118 and 0.275, respectively. The slope factor of perimeter and cooling rate was larger than that of area and cooling rate, which showed that the increase of perimeter had a strong mitigative effect on the internal temperature of the patch, whereas the increase of area did not serve the purpose.

## Discussions

1. By referring to the research of Jia et al.^24^ and Gao et al.^25^, Possible UTC (PUTC) can be increased, which mainly includes bare land and grassland with potential for development. The PUTC area of the study area is 538.27 hm^2^, among which the bare land area is 480.79 hm^2^ and the grassland area is 57.48 hm^2^. The tree canopy coverage rate can be increased by 18.58%, so that the maximum tree canopy coverage rate of 43.82% can be achieved. It is one of the effective ways to increase the canopy coverage of urban forests by making full use of the existing bare land, grassland, and potential tree canopy coverage areas on impermeable surfaces such as parking lots, squares, and sidewalks without tree canopy coverage. In the meantime, the beauty of urban phytocoenosium should be taken into account, some open spaces reserved in an appropriate fashion.
2. Amid the rapid urbanization, urban temperature goes up year by year and the heat island effect aggravates. The cooling effect of tree canopy formed by various urban green spaces and water bodies on the environment is particularly crucial. According to the study findings, as the mean LST of tree canopy and water body is low, for the sake of giving better play to the cooling effect, the future urban planning should attach greater importance to the combination of proper spatial organization and optimal layout among varied land use types. The diversity of the urban landscape patterns should be appropriately augmented, ensuring mutual penetration and uniform distribution of green space, water body and hard landscape. Therefore, the mode of surface thermal radiation effect is altered to generate a stable cold source^26^.
3. Due to the restricted amount of super-large and extra-large patches in the study area, certain conclusions drawn from the quantitative study conducted on the correlation between UTC patches and urban thermal environment may be short of rigorous statistical analysis. At the same time, as the LST was adopted to represent the characteristics of urban thermal environment in the study, there may be some deviations between the LST inversion data retrieved from the remote-sensing images on March 18th, 2021 and the real temperature in the study area. Some other limitations in the aspect of time and space scale should also be considered, for the location being restricted to the urban built-up area of Luoping County. Moreover, further investigations on how to establish a more accurate model of temperature distribution pattern for the majority of areas in various time periods of the year are expected.
4. There is an interaction effect between UTC patches themselves, and with other patches, and the LST inside the tree patches is highly susceptible to the influence of surrounding environment. In this study, only the area, perimeter and shape index of the patch were selected as the independent variables for the influence of the characteristics of the tree canopy patch on the LST inside the patch. For future study, multiple internal and external factors should be taken into account to study the influence of UTC patch on urban thermal environment. In the case that it is difficult to greatly increase the canopy coverage of urban forests, the purpose of regulating the urban thermal environment can be achieved by optimizing the layout of urban forest patches and prioritizing the adjustment of patch characteristics.
5. As a high-density central urban area, urban built-up areas are in short supply of construction land. It is not realistic to increase the area of green space in a large amount. The tree canopy is the key element of the green space system, in future planning, measures such as supplementary increase of tree canopy, protection and expansion of original tree canopy,, reasonable use of the space under the canopy and other measures, from “from the ground to the space, from the green space to the canopy”, a new path for increasing green space in high-density central urban areas. Utilize the limited urban space to improve the efficiency of urban greening such as urban cooling, benefit the lives of citizens, and provide a wide habitat for animals.

## Conclusion

Based on eCongnition Developer 9.0, ENVI 5.3 and Arcgis 10.7 software, the distribution of LST and land cover types in the study area was extracted. The result of the study reveals that the highest LST was 37.63 ℃, the lowest 24.73 ℃, and the mean value 30.83 ℃. Among the land cover types in the study area, buildings and impervious surfaces accounted for the largest proportion and were widely distributed; grassland and water bodies accounted for a smaller proportion. On the strength of the ground temperature distribution of different land cover types, the mean LST of impervious surface was the highest, followed by bare land, grassland, tree canopy, and water body. In this study, 63 patches with super-large and extra-large tree canopy coverage, diversified shapes and distinct cooling effect were selected to investigate the regulatory effect of UTC patches on urban thermal environment. The results revealed that the cooling rate of the patch could go up while the internal temperature of the patch being reduced by means of increasing the area and perimeter of the tree canopy patch. The results of regression analysis on the patch area, perimeter and the cooling rate, after tree canopy patch shape index being eliminated, manifested that the relationship between area and cooling rate was closer than that between perimeter and cooling rate, and the addition of perimeter had a stronger alleviating effect on patch internal temperature, whereas the increase of area had a weaker alleviating effect in this regard.

## Data Availability

The data that support the findings of this study are available from the corresponding author, [PENG Jian-song], upon reasonable request.
